# Parental incubation exchange in a territorial bird species involves sex-specific signalling

**DOI:** 10.1186/s12983-019-0306-0

**Published:** 2019-03-22

**Authors:** Martin Sládeček, Eva Vozabulová, Kateřina Brynychová, Miroslav E. Šálek

**Affiliations:** Faculty of Environmental Sciences, Czech University of Life Sciences Prague, Kamýcká 129, 165 00 Praha, Suchdol Czech Republic

**Keywords:** Biparental incubation, Incubation rhythms, Parental care, Shorebirds, Nest relief, *Vanellus vanellus*, Waders

## Abstract

**Background:**

Effective communication between sexual partners is essential for successful reproduction. Avian parents with biparental incubation need to know how to negotiate, when and who will incubate, and how to harmonize partner exchange at the nest. Although considerable effort has been dedicated to studies of incubation rhythms, few studies have investigated how behavioural signals serve to tighten cooperation between parents. Moreover, existing studies are almost exclusively restricted to species in which long distances between incubating and non-incubating parents prevent continuous communication during incubation. Thus, the most frequently described parental exchange system is a simple model characterized by the return of the non-incubating parent to the nest itself. Here, we propose more complex parental exchange behaviour in the Northern Lapwing (*Vanellus vanellus*), a territorial species capable of continuous partner communication during incubation and with a highly variable male contribution to incubation.

**Results:**

Northern Lapwing females regularly vocalized shortly before departing from the nest, while males mostly left the nest quietly. Responsiveness of the male to female vocalization, perhaps in combination with her flying away from the nest, helped to synchronize incubation care by increasing the probability of exchange, and also by shortening the exchange gaps. In contrast, a male-to-female exchange gap most often occurred after the male quietly flew away from the nest. The frequency of female vocal signalling was not correlated with the male incubation effort on a between-nest scale, but the highest probability of a female-to-male exchange occurred after vocal signalling by females with the most nest-attentive males. Conversely, lowered effort by females to vocalize in the night was accompanied by lower willingness of males to incubate.

**Conclusions:**

Our results suggest that (1) that the incubating parent can communicate with the non-incubating partner using sex-specific behavioural signals, and this helps to synchronize parental exchange on the nest, (2) this signalling may combine acoustic and visual cues, and (3) the efficiency of this signalling might influence the overall nest attendance. The presumption that the repertoire of behavioural signals during reproduction will be much more complex in territorial species that are capable of continuous communication between the partners during the incubation period should be further tested.

**Electronic supplementary material:**

The online version of this article (10.1186/s12983-019-0306-0) contains supplementary material, which is available to authorized users.

## Background

Effective communication between sexual partners is essential for successful reproduction. In biparental species, in particular, acoustic and visual communication between the partners can tackle issues of sexual conflict [1, 2] and also issues of tighter cooperation [3, 4]. In many avian species, both parents take part in incubating the eggs [5], and this increases the demands on communication between incubating and non-incubating partners. A variety of incubation patterns have been described, ranging from exchanges between partners at the nest every few minutes [6] to incubation sessions lasting several weeks [7–9]. However, a question remaining almost unstudied concerns how parents communicate on the scale of particular exchanges.

Most studies targeting the question of partner exchange at the nest have been carried out on species in which the non-incubating parent spends its off-duty time far from the nest [9–12], and thus the parents are unable to communicate continuously. The only feasible way to make a synchronous partner exchange in these cases is therefore probably for the off-duty parent to return to the nest itself [8]. In seabirds, such as albatrosses [9], penguins [13] and skuas [14] with extremely long incubation bouts and hundreds of kilometres long foraging trips, the incubating bird waits until the partner returns. Any failure in this return can therefore lead to a critical decline in the body condition of the incubating bird, and even to abandonment of the nest [7, 8, 11]. However, even in species with much more frequent nest relief, the exchanges usually take place while both parents are present at the nest. This is frequently accompanied by some kind of displays [15] or by other rituals, such as allopreening [3, 16].

There is much more opportunity for communication between the partners and for negotiating about the timing of their exchange on the nest in species where the non-incubating parent spends most of its off-duty time near to the nest, or if it frequently visits the nest even during its off-duty time. Multiple visits preceding an exchange were observed in captive ringed doves (*Streptopelia risoria*) [3]. These regular contacts enable tight cooperation between the parents. Only 13% of nest reliefs were initiated by nest abandonment by the incubating bird before the partner returned. Similarly, in zebra finches (*Taeniopygia guttata*) such regular visits are accompanied by repeated acoustic duets, through which the sitting bird signals its need to be exchanged [4, 17]. In these cases, both birds are probably involved in the negotiation process about when it is time to exchange incubation duties. This can help in achieving tight coordination of incubation care [3, 4, 18].

However, in many species it is not unusual for the incubating parent to leave the nest before the arrival of its partner, and thus the incubation sessions are separated by so-called “exchange gaps” [19, 20]. It is undesirable for the exchange gaps to be too long, because they may increase the risk of nest depredation [21] or cooling of the unattended eggs [22]. Even species that have exchange gaps as a regular part of their incubation schedule should therefore use some request signalling for nest relief. However, the mechanisms for communication between the partners in these species aimed particularly at motivating the non-incubating parent to return to the nest and engage in incubation duties are poorly understood.

The Northern Lapwing (*Vanellus vanellus*) is a biparentally incubating shorebird with a highly variable male contribution to incubation [23–26], and with irregular frequency of parental exchanges [25–27]. The male contribution to incubation is ordinarily smaller than the contribution of the female. The male contribution peaks during the day, while it is almost totally absent in the night [26, 28]. The Lapwing has intermittent incubation, with about 13% of the time when the nest is not attended by either parent [26]. However parental exchange occurs only during a relatively small part of the incubation recesses (Actograms in: [25], this paper). Northern Lapwings are territorial, and the birds spend most of the time in their territories, usually in open habitats [29, 30], which enables continuous contact and communication between partners [30].

In this paper, we analyse behavioural patterns associated with incubation gaps in breeding Northern Lapwings. We hypothesized that the incubating parent communicates with the non-incubating partner using behavioural signals, and that this helps to synchronize parental exchange on the nest. Specifically, and based on our direct observations, we suggest that when intending to exchange with the partner, the incubating parent vocalizes briefly (i.e. for a few seconds) before departing from the nest. The urgency of this signal can be reinforced by flying away from the nest, a more pronounced action than walking away. If this is true, we would expect that 1) partner exchange will occur more probably during the recesses after the departure of the on-duty parent, after issuing a vocalization signal, perhaps reinforced by flying away; 2) there will be shorter recesses accompanied by nest relief coming after these signals (i.e. the signals increase partner synchronization); 3) if the off-duty parent ignores the signal, the subsequent recess will be longer than the recesses without signalling, as a result of partner disagreement within the negotiation process.

Based on the fact that the male contribution to incubation varies strongly among the nests [23–26], we further investigated whether the variation in the male contribution to incubation 1) is predicted by the vocal signalling effort made by the female, or 2) reflects the efficiency of these signals (i.e. more incubating males exchange the female more probably after her signalling). Similarly, because the male contribution to incubation shows strong daily rhythmicity, being highest during the day (with peaks after sunrise and before sunset) and is almost absent in the night [25, 26], we further tested: 3) whether the effort put into signalling by the female changes in the course of the day, and 4) whether the signalling efficiency (i.e. male willingness to exchange) changes in the course of the day.

## Methods

### General field procedure and data extraction

We monitored the incubation of Northern Lapwings in the České Budějovice basin, Doudlebia, Czech Republic (49.25°N, 14.08°E), on approximately 40 km^2^ of agricultural landscape, during April and May 2016. We searched for nests by thoroughly scanning fields and meadows with telescopes, or by walking through areas with high nest densities. We monitored incubation with a small camera (Ø 2 cm, length 4 cm) placed approximately 1.5 m from the nest in a southward direction, in order to minimize the time that the lens faced the sun (which would have overexposed the videos and made individuals hard to recognize). The digital recorder stored videos at 10 frames per second with 640 × 480-pixel resolution. The system was powered by a 12-V, 44-Ah battery buried together with the recorder under the ground. The target was to obtain ~ 2–3 days of recordings from each nest.

We extracted the incubation behaviour using AVS Media Player (http://www.avs4you.com/AVS-Media-Player.aspx). First, we determined each arrival or departure of incubating birds with precision of 1 second. The sex of the birds was determined on the basis of sex-specific plumage traits, such as crest length and the extent of the melanin ornaments on the breast and on the face [31]. Then, we thoroughly scanned the last 5 seconds before each departure in order to identify whether or not the incubating bird had vocalized. Vocalization was clearly identifiable on the videos by specific head movements and by bill opening. As two of the video sets that were used were additionally provided with a small microphone, we were able to validate the linking of specific head and bill movements with vocalization.

For each departure from the nest, we scored vocalization as a binomial variable (1 = at least one call; 0 = without a call), and we noted whether the bird flew away or walked away. Because the recordings from some nests were damaged or ended early due to nest depredation, we excluded from the analysis any nests with less than 10 scored incubation recesses.

We defined an **‘incubation recess’** as any period of time for which the nest was unattended by either of the parents. Subsequently, we classified the incubation recess as a **‘break’** (the same parent came back and continued incubation) or as an **‘exchange gap’** (parents exchanged during the incubation recess) [19]. In order to relate female vocal signalling with the between-nest variation in the male contribution to incubation, we introduced a term **‘male incubation effort’**, calculated as the ratio of male nest attendance at the nest to the overall time for which the nest was attended by either of the parents (i.e. excluding all incubation recesses). **‘Female vocalization effort’** was then defined as the proportion of female departures accompanied by vocalization (per particular nest/hour), and **‘female vocalization efficiency’** was defined as the probability that the male will come to incubate after female vocalization.

### Validation of the assumptions, to avoid confounding effects

In order to correctly interpret the results of this study, we first explored the vocalization pattern of incubating Northern Lapwings with a particular focus on the context of departure from the nest. We investigated whether vocalization can occur frequently at any time during incubation (and might thus confound our interpretation of partner behaviour) or whether it is concentrated just before departure from the nest (as predicted for the purposes of this study). We therefore specifically analysed a subset of 40 nests (~ 960 h) with 1 day of continuous (i.e., completely uninterrupted) videotaping, which enabled us to determine in detail all vocal sessions throughout a one-day incubation course. The set consisted of 30 nests collected in another study in 2015, and a subset of 10 nests from 2016 that were included in this paper.

**Fig. 1:**
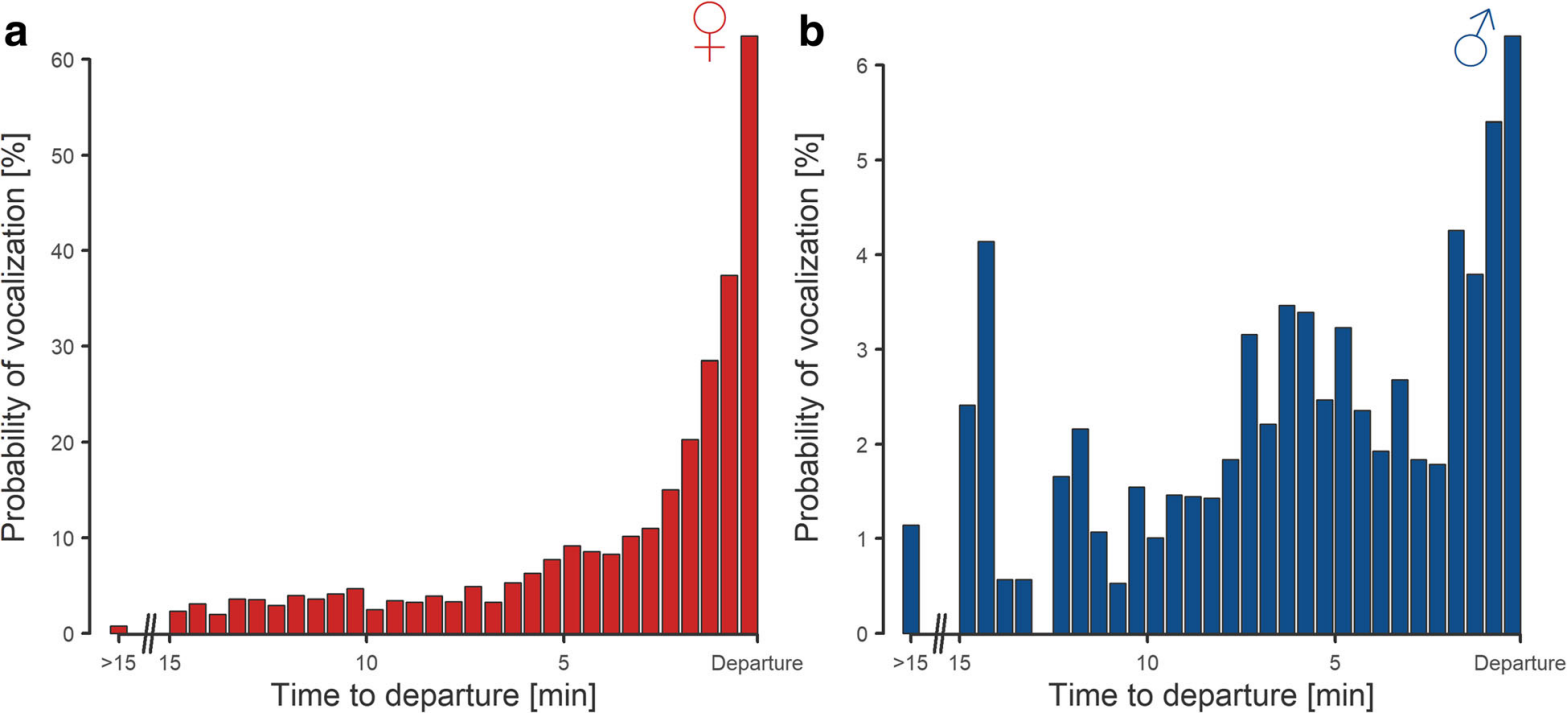
Vocalization in relation to the time prior the end of incubation bout. Bars represent 30 s periods before leaving the nest (departure) and depict the probability that female (**a**; red) or male (**b**; blue) vocalized at least once within a period. The left-most bars (> 15) depict probability of vocalization (mean value per 30 s periods) more than 15 min before the departure. Note that Y-axis range differs between the sexes. Presented data include complete 24-day incubation footages for 40 nests. Ten of these nests are a part of the dataset presented in this paper while other 30 nests used in this figure were collected using the same method in the same area in 2015

We found that although vocalization events could take place at any time during the incubation bouts in both sexes, the frequency steeply increased in few minutes prior to departure. Whereas in males the pattern is weak, in females it is much more pronounced. The vocalization of females peaks immediately before the departure, with more than 60% probability of vocalization during the last 30 s. It contrasts with strongly decreasing probability up to 1.3% (mean probability of vocalization for any thirty-second interval five or more minutes prior to departure; Fig. 1a, b). Secondly, using this dataset, we investigated whether more attentive males (i.e. those that made a greater incubation effort) could have been (positively) assortatively mated with more vocal females, which would confound our interpretation of female vocal signalling efficiency. We observed no positive correlation, and we conclude that the incubation effort in males is not directly positively associated with the vocalization frequency of their female mates (Additional file 1: Figure S1, Table S1).

### Statistical analysis

All statistical analyses were performed in R version 3.3.0 [32]. For the model-based parameter estimates (or for the contrasts between these estimates) we report the effect sizes as medians and Bayesian 95% credible intervals (95%CrI) represented by the 2.5 and 97.5 percentiles from the posterior distribution of 5000 simulated values obtained by the ‘sim’ function from the ‘arm’ R package [33].

Binomial response variables were fitted with generalized mixed-effect models with a binomial error structure and the logit link function, using the “glmer” function from the “lme4” R package [34]. In particular, in order to explain the probability of an exchange gap (i.e. the probability of nest relief during an incubation recess) we used three binomial predictors: “sex”, “vocalization” (yes or no) and “departure type” (“flight” or “walk”). All these effects were used both as main effects and in interactions (including three-way interaction). To explain the probability of vocalization before departure, we also used “sex” and “departure type” as predictors in terms of main effects and in interaction.

In order to test the daily rhythmicity in the female vocalization effort, we also used vocalization before female departure (yes or no) as a response in the model, with time as a predictor. We used time transformed to radians (2*time * π*/*period of interest) and subsequently fitted it as the sine and the cosine of the radians. We used 24 h as a period of interest and, due to the obvious bimodality of the response variable, with peaks in the morning and in the late afternoon, we also used 12 h as a period of interest. Similarly, the “departure type” binomial response was fitted with time (24-h rhythmicity) in interaction with sex.

The length of the incubation recesses was fitted with the mixed-effect model with a Gaussian error structure using the “lmer” function from the “lme4” R package [34]. The response variable was log-transformed to approach the normality of the model residuals. Binomial variables “sex”, “vocalization” (yes or no) and “departure type” (“flight” or “walk”) were used as predictors in the model. We fitted nest identity as a random intercept in all the models described above, and in models using temporal information as a predictor we also fitted time (sine and cosine) as random slopes [35].

To analyse the between-nest differences in female vocalization effort, we used the male incubation effort as a response variable. Female vocalization effort and vocalization efficiency were then z-standardized (centered and mean-divided [36]), and were used as predictors in a general linear model fitted using the “lm” function [32]. The model was weighted by the square-rooted number of analysed female departures from the nest.

Because of the overall scarcity of male incubation in the night (and thus the small sample size of exchange gaps in the night), we were unable to use models to test the night efficiency of female vocalization or the male responsiveness to these signals. We therefore divided all incubation recesses into those started during the dark part of the day (i.e. when the sun was more than 6° below the horizon) and those started during daylight. We then tested 1) whether female vocalization in the night raised the probability of nest relief, and 2) whether the probability that the male would comply with the signalling is the same for both day and night. We tested these hypotheses using the Boschloo test, a technique from a group of unconstrained exact tests for two binomial proportions, which is suitable for use when small expected values occur. This approach using the *p*-value from Fisher’s exact test as a test statistic is explicitly recommended by Mehrotra et al. [37] as convenient in cases of unbalanced designs. In particular, we used the “exact.test” function from the “Exact” R package [38].

## Results

A total of 63 nests were monitored for 2854 h (12 to 116 h; median = 41.37, sd = 18.2) and 5033 nest departures were scored (23 to 242 from particular nests; median = 77, sd = 36.4). Females departed in 3367 cases (66.8%) and males departed in 1666 cases (33.1%). Overall, an exchange gap occurred in 25.6% of incubation recesses (CrI: 22–30%), and was on an average 17% (CrI: 14–20%) more likely after male departures (710 out of 1666; 37.6%; CrI: 34–41%) than after female departures (719 out of 3367; 20.3%; CrI: 17–24%).

### Patterns of nest departures and vocalization

**Fig. 2:**
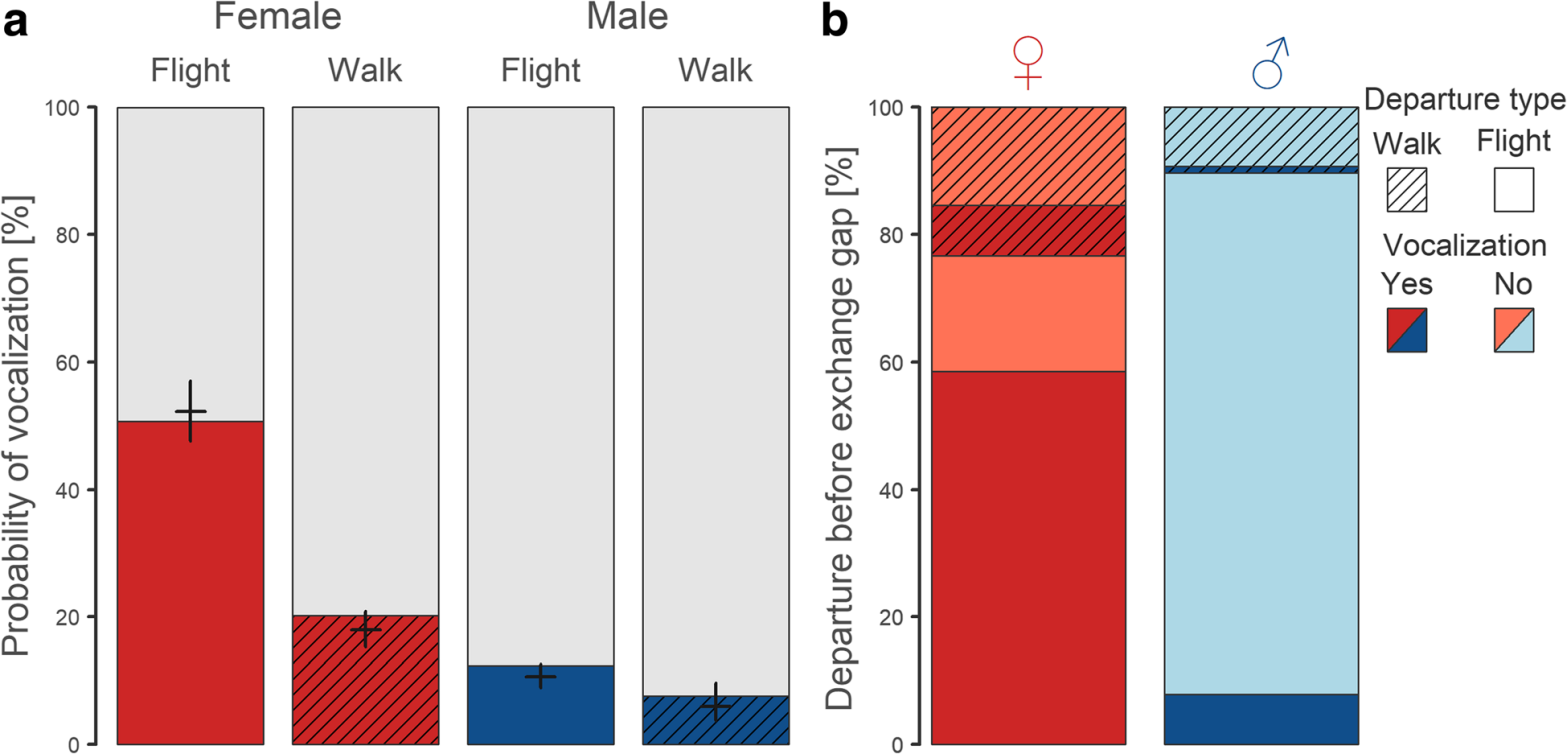
**a** Vocalization in relation to sex and type of a bird’s departure. Bars show the probability of a female (red) and male (blue) vocalization before the bird left the nest by flight (solid bar) or walking (hatched bar). Horizontal lines of black crosses denote estimates from a mixed-effect model with nest identity as a random intercept (Additional file 1: Table S2). The vertical lines denote 95% credible intervals of the estimates. **b** Sex-specific departure type before an exchange gap. Bars represent the relative proportions of exchange gaps (i.e. parents exchanged during the incubation recess) after female (red) and male (blue) incubation bouts with distinction between walk (hatched bars) and flight (solid bars) departures. In addition, dark colours indicate vocalization of a departing bird

**Fig. 3:**
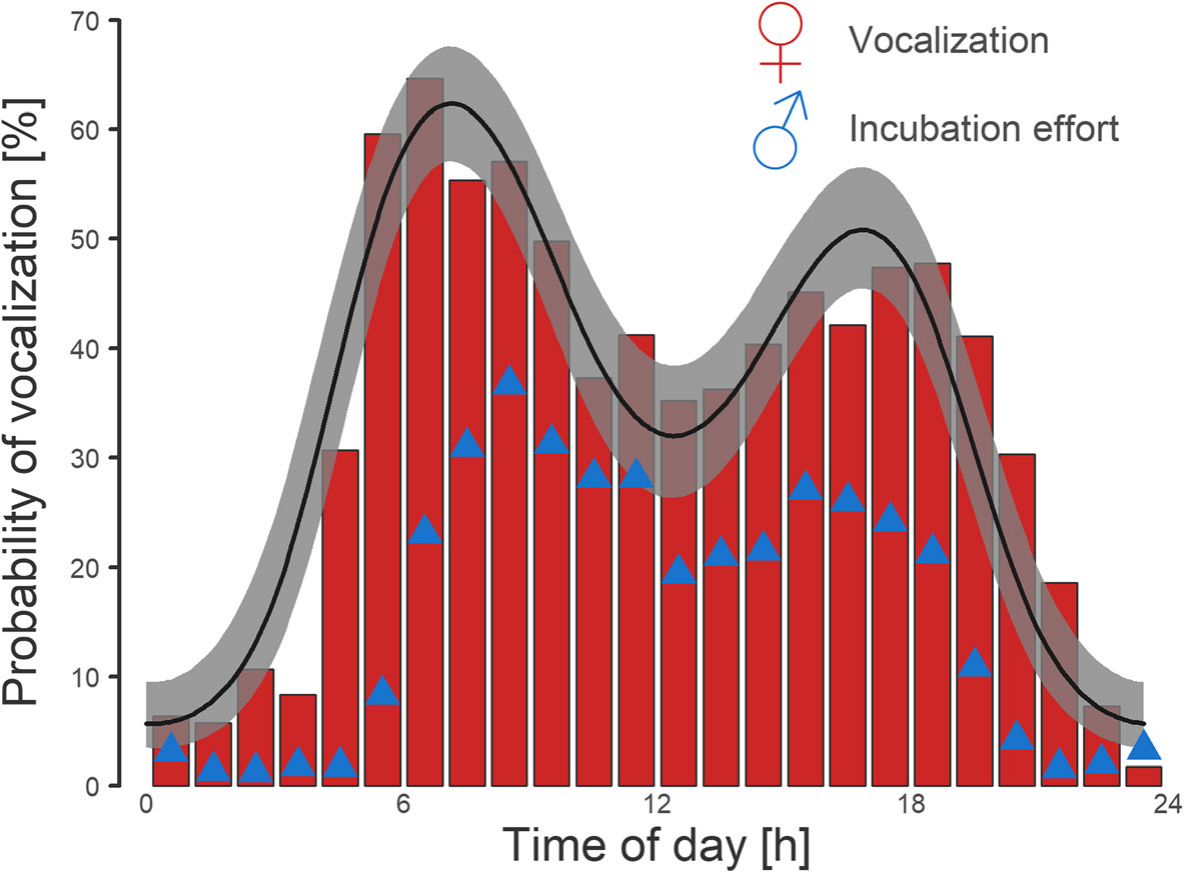
Daily pattern of female vocalization before leaving the nest and male incubation effort. Red bars depict real proportions of female departures accompanied by her vocalization for a particular hour of the day. The curve with shaded area indicates the model prediction with a 95% credible interval (Additional file 1: Table S4). Blue triangles illustrate the proportion of male contribution to incubation in our dataset for a particular hour of day

The use of departure types (flight or walk) and also the probability of vocalization before departure differed between the sexes and varied with the time of day. Males flew away (1415 cases; 87.1% of flight departures; CrI: 84–89%) more often than females (2317 cases; 70.4%; CrI: 67–74%), and females accompanied their departures with vocalization much more often (1385 cases; 41.5%; CrI: 37–46%) than males (193 cases; 10.3%; CrI: 8–12%). Females (but not males) vocalized much more frequently when they flew away from the nest than when they walked away (52 vs. 18%; Fig. 2a, Additional file 1: Table S2). In the daily pattern of females, flight departures prevailed during the night, while they dropped to less than 50% around midday (Additional file 1: Figure S2a, Table S3). In males, this drop was less pronounced, albeit still significant (Additional file 1: Figure S2b, Table S3). The daily pattern of female vocalization during nest departures was bimodal, with peaks after sunrise and before sunset, and followed the ratio of the male contribution to incubation (with the minimum during the night; Fig. 3, Additional file 1: Table S4).

### Probability of exchange gaps with sex-specific signalling

**Fig. 4:**
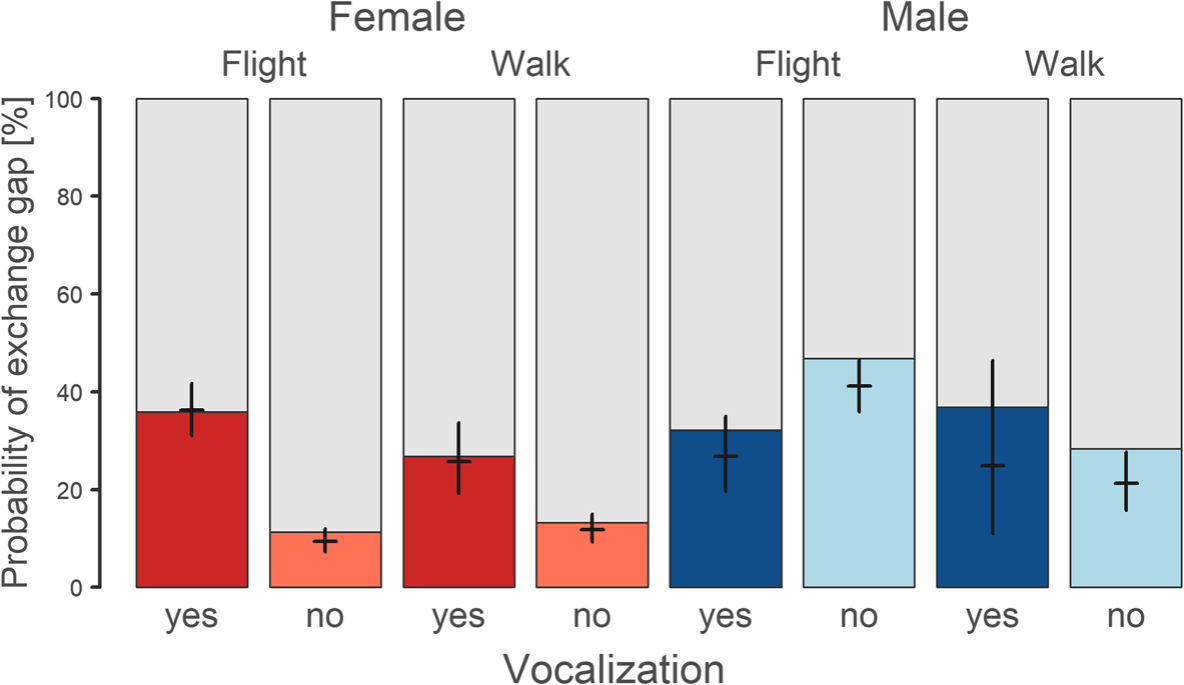
The probability of an exchange gap during an incubation recess. Bars show the probability that a female (red) or male (blue) is exchanged by the partner. Dark colour indicates, that the departing bird vocalized before the departure. Labels above the plot distinguish if the nest was left by flight or walking. The horizontal lines of black crosses denote estimates from the mixed effect model with nest identity as a random intercept (Table 1). The vertical lines indicate 95% credible intervals of the estimates

The probability of parental exchange after an incubation recess was associated with vocalization by an incubating female, but not male. In females, the probability of being exchanged by a male was enhanced by previous vocalization, both when the female flew away (36% vs 9% without vocalization; Fig. 4, Table 1) and when she walked away (26% vs 12% without vocalization; Fig. 4, Table 1). In addition, an exchange after female vocalization was more likely after she flew away than after she walked away (see non-overlapping CrIs in Table 2). Nevertheless, female flight departure itself (i.e. without vocalization) did not increase the probability of an exchange gap. Out of 719 exchange gaps after female incubation, 478 (i.e. 66%, Fig. 2b) were preceded by female vocalization, and of these 421 (58%, Fig. 2b) were also followed by flight departures. In contrast, in males the vocalization before flight departure decreased the probability of male-to-female exchange (Fig. 4, Table 1).

**Table 1:** Probability of exchange gap during incubation recess

					95% CrI	
Level	sex	Vocalization	Type of departure	Estimate	Lower	Upper
1	F	YES	FLIGHT	0.36	0.31	0.42
2	F	NO	FLIGHT	0.09	0.07	0.12
3	F	YES	WALK	0.26	0.19	0.34
4	F	NO	WALK	0.12	0.09	0.15
5	M	YES	FLIGHT	0.27	0.2	0.35
6	M	NO	FLIGHT	0.41	0.36	0.47
7	M	YES	WALK	0.25	0.11	0.47
8	M	NO	WALK	0.21	0.16	0.28

**Table 2:** Probability of exchange gap during incubation recess

		95% CrI	
Contrast	Estimate	Lower	Upper
1–2	**0.27**	**0.22**	**0.31**
1–3	**0.1**	**0.03**	**0.17**
3–4	**0.14**	**0.08**	**0.21**
2–3	**−0.16**	**−0.24**	**−0.1**
2–4	−0.02	− 0.05	0.01
5–6	**−0.14**	**− 0.21**	**− 0.07**
5–7	0.02	− 0.2	0.18
7–8	0.03	−0.11	0.25
6–7	0.16	−0.05	0.3
6–8	**0.2**	**0.14**	**0.25**
1–5	**0.1**	**0.02**	**0.17**
2–6	**−0.32**	**−0.36**	**−0.27**
3–7	0.01	−0.21	0.16
4–8	**−0.09**	**−0.16**	**− 0.04**

The posterior estimates (medians) of the effect sizes with the 95% credible intervals (CrI) from a posterior distribution of 5000 simulated values generated by the ‘sim’ function in R [33]. Variance components were estimated by the ‘glmer’ function for binomial errors with logit link function [34]. **1)** Estimates for particular factor combination levels (see Fig. 4). **2)** Estimates for selected contrasts (number in column “contrast” refers to level number in Table 1). Note that presented values were back-transformed. Those contrasts whose 95% credible intervals do not contain 0 are highlighted in bold

### Effect of vocalization on the synchronization of exchange gaps

**Fig. 5:**
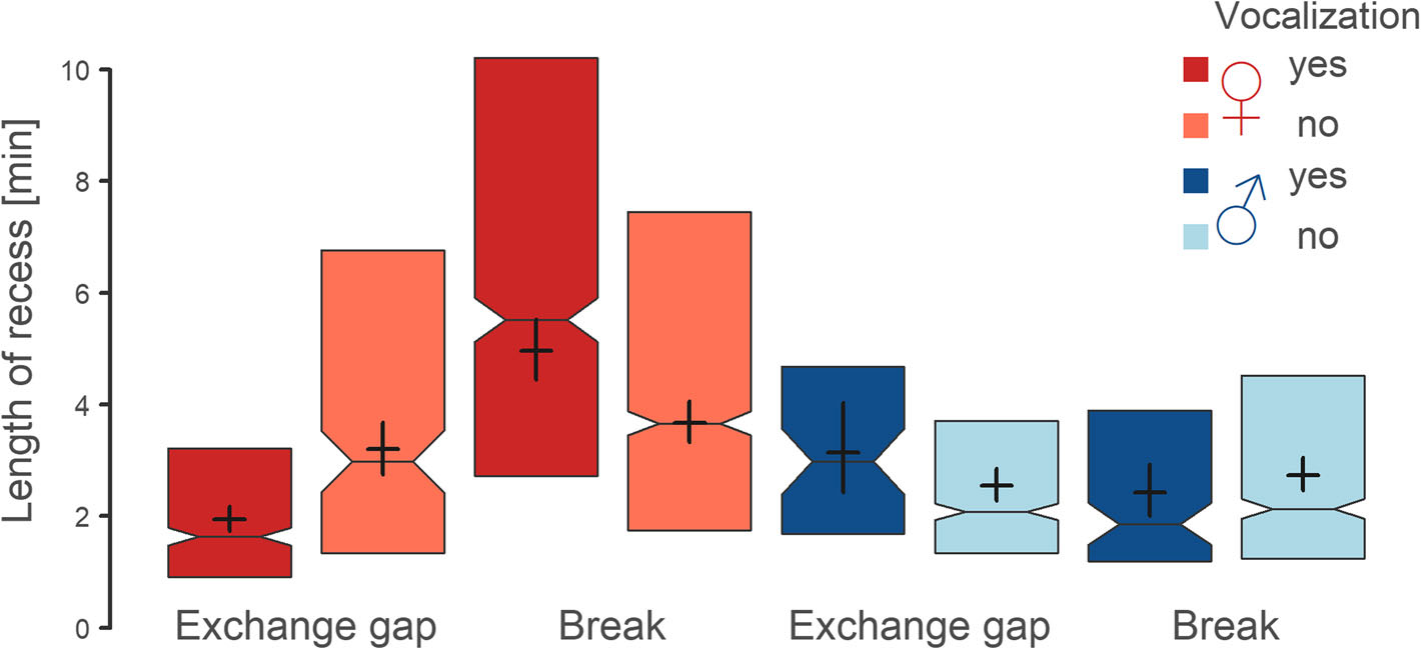
Length of incubation recess in relation to sex, vocalization and type of a bird’s departure. The boxplots summarize lengths of the recesses after female (red) and male (blue) incubation bouts, colour intensity indicates whether the bird vocalized before leaving the nest (dark colours) or did not (light colours). Recesses are classified either as an “Exchange gap” (parents exchanged during the incubation recess) or as a “Break” (the same parent returned and continued incubation). The median length of the recess is depicted by the vertical line inside the box, its 95% confidence interval by the notch, and the 25–75% quantiles by the box. The horizontal lines of black crosses denote estimates from the mixed effect model with nest identity as a random intercept (Table 2). The vertical lines indicate 95% credible intervals of the estimates

Female vocalization before departure from the nest helped to synchronize the exchange gaps, since the exchange gaps coming after female incubation bouts were better synchronized (i.e. they were 1.25 min shorter; CrI: 0.85–1.71 min., Fig. 5) after vocalization than without vocalization. The opposite was true if the recess resulted only in a break (i.e. if the male did not come to exchange the female). The breaks coming after female departure accompanied by vocalization were 1.29 min longer (CrI: 0.93–1.68 min.) than those without vocalization (Fig. 5, Tables 3 and 4). Conversely, the incubation recesses of males were generally shorter than those of females, and the length of the exchange gaps coming after male incubation bouts was not affected by whether or not the male vocalized.

**Table 3:** Length of recess

					95% CrI	
Level	sex	Vocalization	Type of gap	Estimate	Lower	Upper
1	F	YES	EXCHANGE	1.94	1.74	2.17
2	F	NO	EXCHANGE	3.19	2.75	3.7
3	F	YES	RECESS	4.96	4.42	5.52
4	F	NO	RECESS	3.67	3.33	4.04
5	M	YES	EXCHANGE	3.15	2.45	4.03
6	M	NO	EXCHANGE	2.54	2.28	2.86
7	M	YES	RECESS	2.42	2.02	2.93
8	M	NO	RECESS	2.73	2.45	3.05

**Table 4:** Length of recess

		95% CrI	
Contrast	Estimate	Lower	Upper
1–2	**− 1.25**	**−1.71**	**−0.85**
1–3	**−3.02**	**− 3.46**	**− 2.6**
3–4	**1.29**	**0.93**	**1.68**
2–4	**−0.48**	**−0.88**	**− 0.03**
5–6	0.6	−0.1	1.47
5–7	0.72	−0.07	1.65
7–8	−0.31	−0.74	0.17
6–8	−0.19	−0.46	0.07
1–5	**−1.21**	**− 2.05**	**−0.52**
2–6	**0.65**	**0.23**	**1.11**
3–7	**2.53**	**1.96**	**3.1**
4–8	**0.93**	**0.67**	**1.21**

The posterior estimates (medians) of the effect sizes with the 95% credible intervals (CI) from a posterior distribution of 5000 simulated values generated by the ‘sim’ function in R [33]. Variance components were estimated by the ‘lmer’ function in R [34]. **3)** Estimates for particular factor combination levels (see Fig. 5). 4**)** Estimates for selected contrasts (number in column “contrast” refers to level number in Table 3). Note that response variable was log-transformed in the model, but presented values were back-transformed. Those contrasts whose 95% credible intervals do not contain 0 are highlighted in bold

**Fig. 6:**
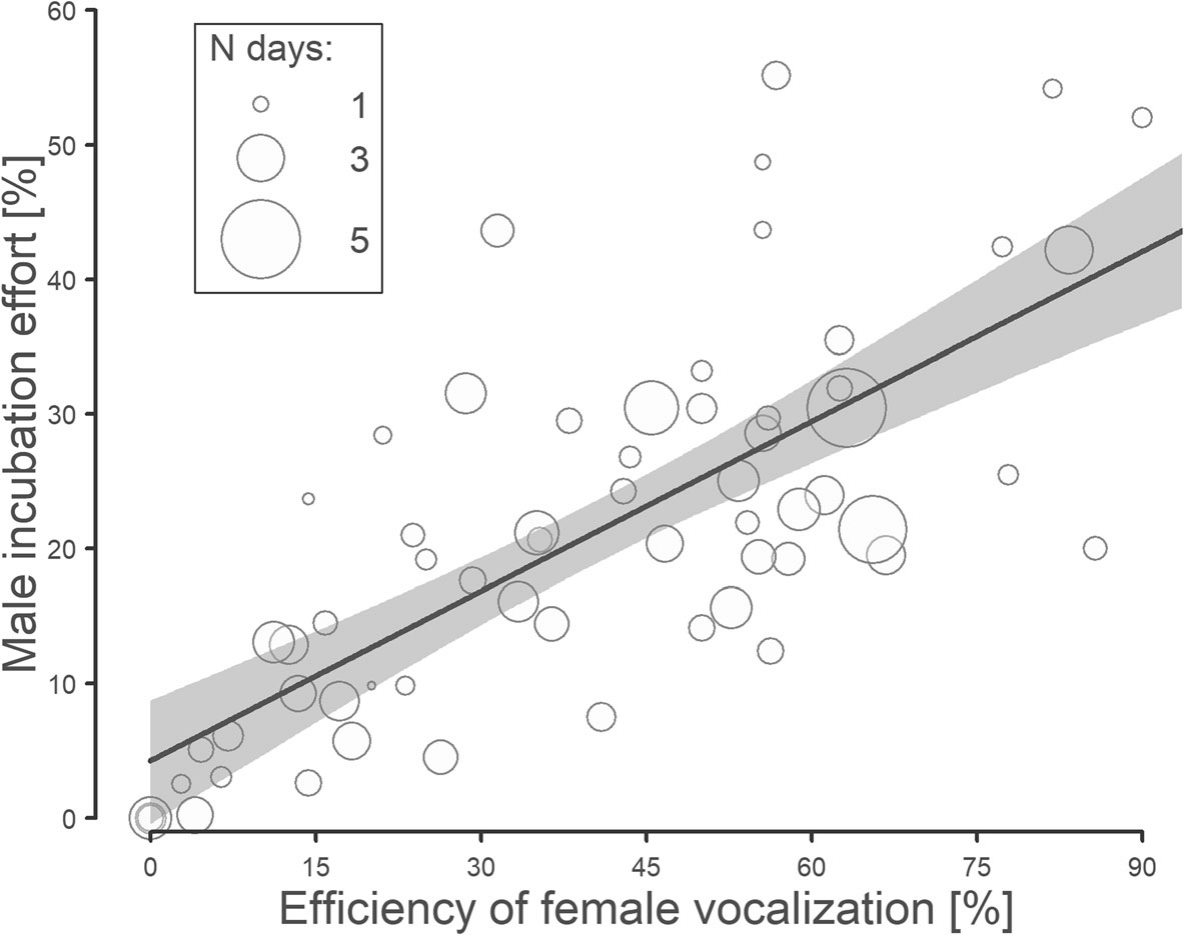
The relationship between male incubation effort and the efficiency of female vocalization. The male incubation effort is taken as the ratio of male nest attendance at the nest to the overall time, for which the nest was attended by either of the parents. The efficiency of female vocalization is the proportion of female departures accompanied by her vocalization after which the male came to incubate (i.e. “Exchange gap” took place). Circles represent the individual nests and their size the number of days with incubation data. The line with shaded area indicates the model prediction with a 95% credible interval (Additional file 1: Table S5)

On a between-nest scale, the male contribution to incubation in a particular nest was not enhanced by the female vocalization effort (i.e. the proportion of departures accompanied by vocalization per particular nest/hour). However, in nests with a higher male contribution to incubation, the males were more likely to come and incubate after female vocalization (Fig. 6, Additional file 1: Table S5).

### Daily pattern in vocalization efficiency

Although the overall frequency of female vocalization in the night was very low (10.7% of departures) and there were only 17 subsequent exchange gaps from 8 nests, female vocalization before departure strongly increased the probability of her being exchanged even in the night (Boschloo test; *p* < 0.001). Nonetheless, the efficiency of female vocalization signalling (i.e. the probability that a male will come after female vocalization) was significantly lower in the night than in daytime (Boschloo test; *p* = 0.017).

## Discussion

In this study, we have revealed several aspects of partner communication in the Northern Lapwing during the incubation period: 1) females (but not males) combine acoustic and motion signals in an attempt to ask the partner for nest relief, and these signals, together with male willingness to exchange with the female, shape the length of the incubation recesses; 2) scarcity of male incubation at night is associated with a lower female vocalization effort, and also with lower male readiness to incubate; 3) the between-nest differences in male incubation effort are shaped by the willingness of the male to provide nest relief, rather than by the female vocalization effort. We discuss these topics below.

### Use of signals

Unlike many other related species with biparental incubation [19, 39, 40], Northern Lapwings have an incubation rhythm that is characterized by frequent but relatively short incubation recesses, only a minority of which (i.e. 25% in our sample) serve as an exchange gap (Fig. 4). Some of the incubation recesses without nest relief therefore have other functions, e.g. leaving the nest unattended during a disturbance or a predator approach (and relying on nest crypsis) [41], a direct predator attack [41, 42], or just a short foraging break. For example, females often took a break around the noon, walked and foraged nearby the nest (our direct observations both in video recordings and in the field).

In addition to the reasons mentioned above, we suggest that a proportion of incubation breaks can also result from failures of the negotiation process about partner exchange at the nest [3]. We show that females had a far higher probability of being exchanged by a male when they vocalized shortly before departing from the nest, and this pattern was more obvious when the female flew away (though the pattern could still be observed when she walked away). This suggests that female vocalization could serve as a signal to the male partner requesting an exchange of incubation duties. The patterns in the length of incubation recesses were also consistent with our predictions; exchange gaps were shortened (i.e. better synchronized) whereas breaks (i.e. recesses without parent exchange) were prolonged when there was female vocalization. Thus, we can assume that when the male does not fulfil the female’s exchange request, the female waits within the negotiation process for a considerably longer period, then returns to continue in incubation.

However, an alternative explanation can be put forward, at least in some events, i.e. that prolonged breaks after female vocalization can occur in cases when the female signals a perceived danger, such as an approaching predator, rather than a need to be exchanged. At the same time, the voice activity of the female often graduates for several minutes before she leaves the nest (see Fig. 1a), and such conspicuous behaviour in the presence of a predator could be counterproductive in terms of nest protection. Moreover, long female breaks after a disturbance (accompanied by vocalization), contrasting with really short female-to-male exchange gaps on the nest in the same situations, seem to be cumbersome and difficult to explain (Fig. 5). Finally, it seems improbable that there would be a rapid female-to-male exchange after a disturbance when the male-to-female exchange is slower, in a species where the main role of a male is to protect the territory from predators and the male participates considerably less than the female in incubation care (Fig. 5). There is a need for further studies to determine the roles of both alternatives suggested here, and their effects on the length of incubation recesses in avian incubation.

We documented also a considerable proportion of exchange gaps (33%; Fig. 2b) after female departure without previous vocalization. We cannot rule out that vocalization occurred in these cases immediately after leaving the nest, when the female was already out of camera view. On the other hand, it might indicate that the negotiation process also involves other signals, made away from the nest, but note that these exchanges were generally worse coordinated (Fig. 5). Some less common alternative ways of communicating, or failures of usual patterns regarding the exchange process, could exist in the Northern Lapwing, as is also found in other species. For example, although regular nest reliefs in Ringed Doves and Herring gulls *(Larus argentatus)* take place in the presence of both parents on the nest, it has been documented that some smaller proportion of the nest reliefs in these species are accompanied by exchange gaps [3, 20], even though such exchange gaps can be accompanied by a severely enhanced risk of egg depredation [21].

We observed different signalling patterns in males than in females. Vocalization was observed in only 11.6% of males, and was even accompanied by a decrease in the probability of an exchange gap. We suggest several possible explanations for this different pattern. Firstly, males may not need any specific requesting signal to negotiate an exchange with the female partner. As parental exchange occurs much more often after male departure than after female departure, the departure of a male who generally incubates less than the female can itself serve as a signal for the female to negotiate an exchange, even without a male call. Furthermore, Lapwing male acoustic signalling during incubation may serve primarily as a warning in response to an approaching predator [43]. We know that Lapwings avoid incubating in the presence of a predator, leaving the nest for the necessary period of time and relying on egg crypsis [41]. The male behaviour described here may therefore be seen as an aspect of the key role of the male in guarding the nest against predators. This could explain why males more frequent fly away from the nest than walk away from it, which would enable the male to attack the predator faster and more effectively [43].

Our findings could suggest that, in contrast with most of the previously studied species [3, 4, 7, 12, 20], the timing of nest reliefs in Northern Lapwings might be induced by the bird that is currently incubating, particularly by females. However, revealing who really initiates the exchange on the nest would require simultaneous recording of both partners (on the nest and away from it), which is a topic requiring further observational research.

### Night incubation

Females greatly lowered their vocalization effort before departing from the nest in the night. This could be because male incubation in the night is very rare in the Northern Lapwing [25, 28, 44], and thus the possibility of being exchanged can be negligible for a female. However, despite the overall scarcity of male night incubation in our sample (17 cases), the probability of an exchange gap after female vocalization during nest departure was still almost 20% (in comparison with 35% during the day), while it was reduced to only 1.6% after a “silent departure” (in comparison with 15% during the day). Thus, although the males showed significantly lowered willingness to provide night nest relief, there was still a substantial chance for a female to get male help on the nest in the night after vocalization signalling.

So, why did the females lower their vocalization efforts so much in the night? We suggest that this pattern could mirror the response to increased predation pressure during the night, when mammalian predators are most active ([45, 46]; own observation). This explanation is justified by the observation that the nests of Northern Lapwings are depredated almost solely by nocturnal mammals ([47]; all 11 cases of known depredations in the study population). Firstly, vocalization during the night can attract nest predators, and females may face a trade-off between sitting quietly for most of the night and loudly highlighting the position of her nest. Our results indicate that most females probably prefer to bear the incubation bout for a whole night in order to be as inconspicuous as possible. Secondly, it could be more beneficial for females to leave the vigilant males to guard the nest in the night, rather than to ask for exchange. In future research, we therefore propose to test the significance of acoustic cues, such as bird calling, on mammal predator orientation in the night. We also need to describe Northern Lapwing male behaviour in the night, with respect to their ability to warn the sitting female about the approach of a predator, which is a strong characteristic feature of Lapwing males during the day [29, 42].

### Between-nest differences in male incubation attendance

As can be found elsewhere [24, 26, 28], the male contribution to incubation is a strong predictor of overall nest attendance in the Northern Lapwing. This could be because of female energy limitations to fully compensate reduced male care [48], or it could be a result of negotiations over parental care [1]. Predictions from theoretical models assume that an evolutionarily stable strategy in response to the reduced parental effort of one partner is for the other partner to compensate to some extent ([1, 49, 50], but see: [51]). This explanation has also been supported by empirical data [52, 53]. Our study suggests a possible extending of this previous knowledge with a new finding in the behaviour of partners in this mechanism: it was found that better incubating males were more willing to come and incubate after the female had signalled her departure from the nest, but that the female signalling effort itself did not affect the extent of male care in a particular nest. This finding, together with the fact that the subsequent recess is longer if a female “exchange request” is not fulfilled by the male, suggests that it is the negotiation process associated with the fine-tuning between the partners that can influence the total nest attendance, rather than an energetic constraint [1]. On the basis of our data, we are not able to quantify the importance of this partnership mechanism and to compare it with the effect of energetic constraints. However, the negotiation process resulting from tuning and compliance between the partners appears to be a possible proximate mechanism that modifies the overall incubation attendance in biparentally nesting birds.

## Conclusion

To conclude, we have documented that, in a territorial species capable of continuous communication between the partners during incubation, vocal and motion signals could be used for better synchronization of nest relief. Because it seems that the effectiveness in negotiating about exchanging parental duties influences the length of incubation recesses, we have also suggested how the negotiation process could influence overall nest attendance. Since we found vocalization signalling only in females, we suggest that behavioural signals serving parental cooperation and negotiation in birds can be sex-specific.

## Additional file

Additional file 1: **Figure S1.** Frequency of female hourly vocalization in relation to male incubation effort. **Figure S2.** Daily pattern of flight away from the nest during a bird’s departure. **Table S1.** The relationship between male incubation attendance and female vocalization effort during the incubation. **Table S2.** Patterns of probability of vocalization**. Table S3.** The probability of flight away during departure. **Table S4.** Circadian pattern of female exchange requesting. **Table S5.** Between nest differences in male contribution to incubation. (DOCX 132 kb)

## References

[1] Lessells CM, McNamara JM (2012). Sexual conflict over parental investment in repeated bouts: negotiation reduces overall care. Proc R Soc B Biol Sci.

[2] Székely T, Kosztolányi A, Küpper C, Thomas GH (2007). Sexual conflict over parental care : a case study of shorebirds. J Ornithol.

[3] Ball GF, Silver R (1983). Timing of incubation bouts by ring doves (*Streptopelia risoria*). J Comp Psychol.

[4] Boucaud ICA, Perez EC, Ramos LS, Griffith SC, Vignal C (2017). Acoustic communication in zebra finches signals when mates will take turns with parental duties. Behav Ecol.

[5] Deeming C (2002). Avian incubation: behaviour, environment and evolution.

[6] Schwagmeyer PL, Bartlett TL, Schwabl HG (2008). Dynamics of house sparrow biparental care: what contexts trigger partial compensation?. Ethology..

[7] Yorio PM, Boersma PD (1994). Causes of nest nesertion during nncubation in the Magellanic penguin (Spheniscus magellanicus). Condor..

[8] Davis L (1982). Timing of nest relief and its effect on breeding success in Adélie penguins (*Pygoscelis adeliae*). Condor..

[9] Weimerskirch H (1995). Regulation of foraging trips and incubation routine in male and female wandering albatrosses. Oecologia..

[10] Bulla M, Stich E, Valcu M, Kempenaers B (2015). Off-nest behaviour in a biparentally incubating shorebird varies with sex, time of day and weather. Ibis..

[11] Chaurand T, Weimerskirch H (1994). Incubation routine, body mass regulation and egg neglect in the blue petrel Halobaena caerulea. Ibis..

[12] Seddon P (1989). Patterns of nest relief during incubation, and incubation period variability in the yellow-eyed penguin (*Megadyptes antipodes*). New Zeal J Zool.

[13] Guinet C, Koudil M, Bost CA, Durbec JP, Georges JY, Mouchot MC (1997). Foraging behaviour of satellite-tracked king penguins in relation to sea-surface temperatures obtained by satellite telemetry at Crozet Archipelago, a study during three austral summers. Mar Ecol Prog Ser.

[14] Jakubas D, Iliszko LM, Strøm H, Helgason HH, Stempniewicz L. Flexibility of foraging strategies of the great skua *Stercorarius skua* breeding in the largest colony in the Barents Sea region. Front Zool. 2018;15(1):–14. 10.1186/s12983-018-0257-x PMC5865383 29588648

[15] Wachtmeister CA (2001). Display in monogamous pairs: a review of empirical data and evolutionary explanations. Anim Behav.

[16] Takahashi LS, Storey AE, Wilhelm SI, Walsh CJ (2017). Turn-taking ceremonies in a colonial seabird: does behavioral variation signal individual condition?. Auk..

[17] Boucaud ICA, Aguirre Smith MLN, Valère PA, Vignal C (2016). Incubating females signal their needs during intrapair vocal communication at the nest: a feeding experiment in great tits. Anim Behav.

[18] Boucaud ICA, Mariette MM, Villain AS, Vignal C (2016). Vocal negotiation over parental care? Acoustic communication at the nest predicts partners’ incubation share. Biol J Linn Soc.

[19] Bulla M, Valcu M, Rutten AL, Kempenaers B (2014). Biparental incubation patterns in a high-Arctic breeding shorebird: how do pairs divide their duties?. Behav Ecol.

[20] Niebuhr V, McFarland D (1983). Nest-relief behaviour in the herring gull. Anim Behav.

[21] Ronconi RA, Hipfner JM (2009). Egg neglect under risk of predation in Cassin’s auklet (Ptychoramphus aleuticus ). Can J Zool.

[22] Lislevand T (2001). Male incubation in northern lapwings : effects on egg temperature and potential benefits to females. Ornis Fenn.

[23] Liker A, Székely T (1999). Mating pattern and mate choice in the lapwing *Vanellus vanellus*. Zoology..

[24] Grønstøl GB (2003). Mate-sharing costs in polygynous northern lapwings *Vanellus vanellus*. Ibis..

[25] Sládeček M, Bulla M. Supporting information for “diverse incubation rhythm in a facultatively uniparental shorebird - the northern lapwing.” Open Sci Framew [Internet] 2018;http://osf.io/y4vpe. Available from: http://osf.io/y4vpe 10.1038/s41598-019-41223-z PMC6423287 30886196

[26] Sládeček M, Vozabulová E, Šálek M, Bulla M. Diverse incubation rhythms in a facultatively uniparental shorebird – the northern lapwing. BioRxiv. 2018:324426. 10.1038/s41598-019-41223-z PMC6423287 30886196

[27] Bulla M, Valcu M, Dokter AM, Dondua AG, Kosztolányi A, Rutten AL (2016). Unexpected diversity in socially synchronized rhythms of shorebirds. Nature..

[28] Jongbloed F, Schekkerman H, Teunissen W (2006). Verdeling van de broedinspanning bij Kieviten. Limosa..

[29] Liker A, Székely T (1999). Parental behaviour in the lapwing *Vanellus vanellus*. Ibis..

[30] Lislevand T, Byrkjedal I (2004). Incubation behaviour in male northern lapwing *Vanellus vanellus* in relation to mating opportunities and female body condition. Ardea..

[31] Meissner W, Wójcik C, Pinchuk P, Karlionova N (2013). Ageing and sexing the northern lapwing Vanellus vanellus. Wader Study Gr Bull.

[32] R-Core-Team. R: a language and environment for statistical computing. [internet]. R Foundation for statistical computing; 2017. Available from: http://www.r-project.org/.

[33] Gelman A, Su Y-S, Yajima M, Hill J, Pittau M, Kerman J, et al. Data analysis using regression and multilevel/hierarchical models [internet]. CRAN Repos 2016. p. 1–53. Available from: https://cran.r-project.org/package=arm.

[34] Bates D, Maechler M, Bolker B, Walker S, Christensen RHB, Singmann H, et al. Fitting linear mixed-effects models using lme4. J Stat Softw [Internet] 2015;67:1–48. Available from: https://www.jstatsoft.org/article/view/v067i01

[35] Schielzeth H, Forstmeier W (2009). Conclusions beyond support: overconfident estimates in mixed models. Behav Ecol.

[36] Schielzeth H. Simple means to improve the interpretability of regression coefficients. Methods Ecol Evol [Internet] 2010;1:103–13. Available from: http://doi.wiley.com/10.1111/j.2041-210X.2010.00012.x

[37] Mehrotra D V, Chan ISF, Berger RL. A cautionary note on exact unconditional inference for a difference between two independent binomial proportions. Biometrics [Internet] 2003;59:441–50. Available from: http://www.ncbi.nlm.nih.gov/pubmed/12926729 10.1111/1541-0420.00051 12926729

[38] Calhoun P. Exact: Unconditional Exact Test. R package version 1.6. 2015; Available from: https://cran.r-project.org/package=Exact.

[39] Bulla M, Valcu M, Dokter AM, Dondua AG, Kosztolányi A, Rutten AL, et al. Supporting information for “unexpected diversity in socially synchronized rhythms of shorebirds.” open Sci Framew [internet]. Open Science Framework; 2016;10.17605/OSF.IO/WXUFM. Available from: 10.17605/OSF.IO/WXUFM 10.1038/nature20563 27880762

[40] Pedler RD, Weston MA, Bennett ATD (2015). Long incubation bouts and biparental incubation in the nomadic banded stilt. Emu..

[41] Šálek M, Cepáková E (2006). Do northern lapwings *Vanellus vanellus* and little ringed plovers *Charadrius dubius* rely on egg crypsis during incubation?. Folia Zool.

[42] Kis J, Liker A, Székely T (2000). Nest defence by lapwings: observation on natural behaviour and an experiment. Ardea..

[43] Cramp S, Simmons KL (1983). Handbook of the birds of Europe, the Middle East and North Africa. The birds of the Western Palearctic: 3. Waders to gulls. Cramp S, Simmons KL., editors.

[44] Lislevand T, Byrkjedal I, Grønstøl GB, Hafsmo JE, Kallestad GR, Larsen VA (2004). Incubation behaviour in northern lapwings: nocturnal nest attentiveness and possible importance of individual breeding quality. Ethology..

[45] Weidinger K (2010). Foraging behaviour of nest predators at open-cup nests of woodland passerines. J Ornithol.

[46] Praus L, Weidinger K (2010). Predators and nest success of sky larks *Alauda arvensis* in large arable fields in the Czech Republic. Bird Study.

[47] Teunissen W, Schekkerman H, Willems F, Majoor F. Identifying predators of eggs and chicks of lapwing *Vanellus vanellus* and black-tailed godwit *Limosa limosa* in the Netherlands and the importance of predation on wader reproductive output. Ibis (Lond 1859). 2008;150:74–85.

[48] Matysioková B, Remeš V (2014). The importance of having a partner: male help releases females from time limitation during incubation in birds. Front Zool.

[49] McNamara JM, Gasson CE, Houston AI (1999). Incorporating rules for responding into evolutionary games. Nature..

[50] Houston AI, Székely T, McNamara JM. Conflict between parents over care. Trends Ecol Evol. 2005:33–8. 10.1016/j.tree.2004.10.008 16701338

[51] Jones K, Ruxton G, Monaghan P (2002). Model parents: is full compensation for reduced partner nest attendance compatible with stable biparental care?. Behav Ecol.

[52] Bulla M, Valcu M, Rutten AL, Kempenaers B. Temporary mate removal during incubation leads to variable compensation in a biparental shorebird. bioRxiv [Internet] 2017;117036. Available from: 10.1101/117036

[53] Bulla M, Valcu M, Prüter H, Vitnerová H, Tijsen W, Sládeček M (2017). Flexible parental care: uniparental incubation in biparentally incubating shorebirds. Sci Rep.

