# Supplementary material for: Parental incubation exchange in a territorial bird species involves sex-specific signalling
Source: Front Zool. 2019 Mar 22;16:7. doi: 10.1186/s12983-019-0306-0 (PMC6431054; doi:10.1186/s12983-019-0306-0)
Supplement: Supplementary file 1 — Figure S1. Frequency of female hourly vocalization in relation to male incubation effort. Figure S2. Daily pattern of flight away from the nest during a bird’s departure. Table S1. The relationship between male incubation attendance and female vocalization effort during the incubation. Table S2. Patterns of probability of vocalization. Table S3. The probability of flight away during departure. Table S4. Circadian pattern of female exchange requesting. Table S5. Between nest differences in male contribution to incubation. (DOCX 132 kb) [file 12983_2019_306_MOESM1_ESM.docx]

**SUPPLEMENTARY INFORMATION**

**Figure S1.**


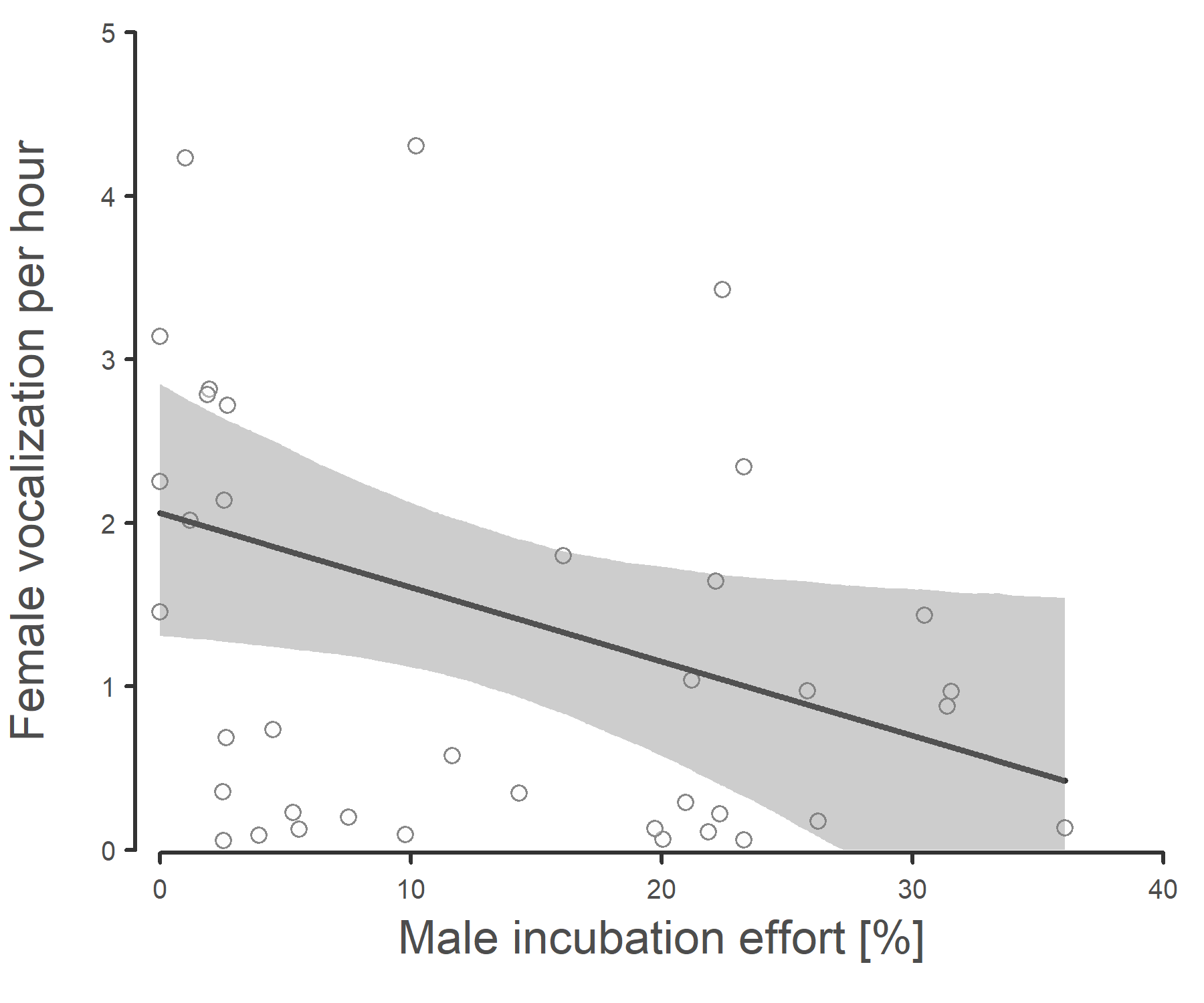


**Figure S1 Frequency of female hourly vocalization in relation to male incubation effort.** Male incubation is taken as the proportion of male nest attendance from the overall time for which the nest was attended by any parent (i.e. excluding all incubation recesses). Frequency of female vocalization is a mean number of female “vocal sessions” per hour of female incubation. Calls separated by at least 30 seconds of silence are considered as two discrete sessions. Presented data include complete 24h-day of incubation footage for 40 nests. Ten out of these nests are a part of other data presented in this paper, while 30 of them were recorded using the same method within the same area in 2015. Line with shaded area indicates the model prediction with 95% credible intervals (Tab. S1). Circles represent the individual nests.

**Figure S2.**


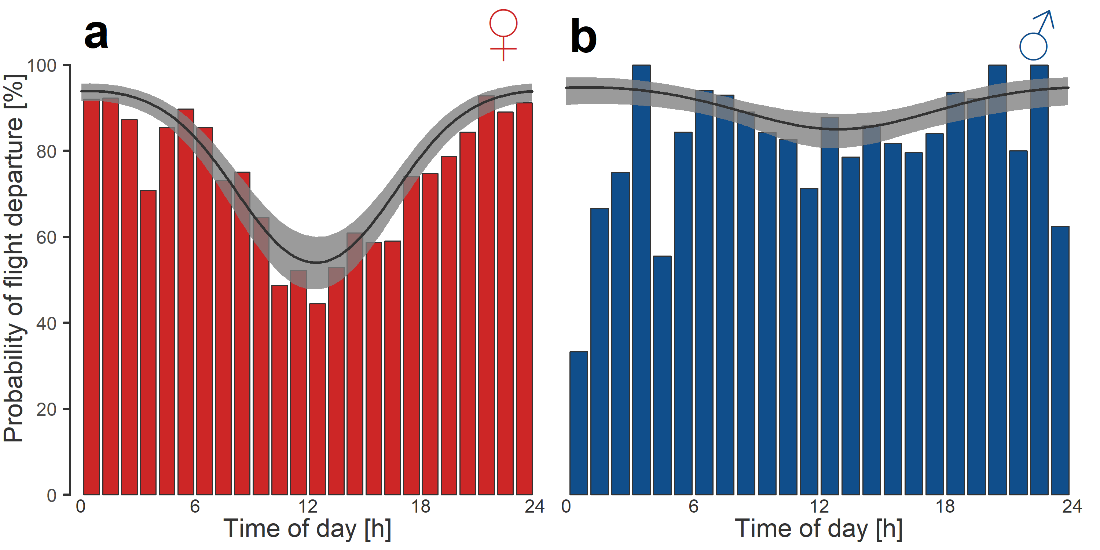


**Figure S2 Daily pattern of flight away from the nest during a bird’s departure.** Bars depict the real probability that female (red) or male (blue) left the nest by flight (i.e. not by walking away), within a particular hour. Lines with shaded areas indicate the model prediction with 95% credible intervals (Table S3).

**Supplementary table S1** | **The relationship between male incubation attendance and female vocalization effort during the incubation.**

|  |  |  | 95% CrI | |
| --- | --- | --- | --- | --- |
| Response | Effect | Estimate | Lower | Upper |
| Female vocalization effort | Intercept | **2,075** | **1,319** | **2,863** |
|  | Male incubation effort | **-4,695** | **-9,332** | **-0,05** |

The posterior estimates (medians) of the effect sizes with the 95% credible intervals (CrI) from a posterior distribution of 5,000 simulated values generated by the ‘sim’ function in R[1]. Variance components were estimated by the ‘lm’ function in R. Estimates whose 95% credible intervals do not contain 0 are highlighted in bold. Male incubation is taken as the proportion of male nest attendance at the nest to overall time for which the nest was attended by any parent (i.e. excluding all incubation recesses). Female vocalization is then taken as mean number of female “vocal sessions” per hour of female incubation. As two discrete vocal sessions are taken the calls separated by at least 30 s without calling. Presented data include complete 24 day incubation footage for 40 nests. 10 out of these nests are also a part of other data presented in this paper, while 30 of them were recorded with the same method and within the same area during 2015.

**Supplementary table S2** | **Patterns of probability of vocalization**

**a**

|  |  |  |  | 95% CrI | |
| --- | --- | --- | --- | --- | --- |
| Level | sex | Type of departure | Estimate | Lower | Upper |
| 1 | F | FLIGHT | 0.52 | 0.48 | 0.57 |
| 2 | F | WALK | 0.18 | 0.15 | 0.21 |
| 3 | M | FLIGHT | 0.11 | 0.09 | 0.13 |
| 4 | M | WALK | 0.06 | 0.04 | 0.1 |

**b**

|  |  | 95% CrI | |
| --- | --- | --- | --- |
| Contrast | Estimate | Lower | Upper |
| 1-2 | **0.34** | **0.31** | **0.38** |
| 1-3 | **0.42** | **0.38** | **0.46** |
| 3-4 | **0.05** | **0.01** | **0.08** |
| 2-4 | **0.12** | **0.08** | **0.16** |

The posterior estimates (medians) of the effect sizes with the 95% credible intervals (CrI) from a posterior distribution of 5,000 simulated values generated by the ‘sim’ function in R[1]. Variance components were estimated by the ‘glmer’ function for binomial errors with logit link function and nest identity as a random intercept [2]. **a)** Estimates for particular factor levels combinations (see Fig. 1a). **b)** Estimates for selected contrasts (number in column “contrast” refers to level number in Table 2a). Note that presented values were back-transformed; contrasts whose 95% credible intervals do not contain 0 are highlighted in bold.

**Supplementary table S3**| **The probability of flight away during departure**

|  |  |  | |  | 95% CrI | |
| --- | --- | --- | --- | --- | --- | --- |
| Response | Effect type | Effect | | Estimate | Lower | Upper |
| Probability of flight departure | Fixed | Intercept | 1.450 | | 1.264 | 1.635 |
|  |  | Sex (M) | 0.870 | | 0.598 | 1.143 |
|  |  | Sin (24 time) | 0.171 | | 0.022 | 0.328 |
|  |  | Cos (24 time) | 1.278 | | 1.029 | 1.519 |
|  |  | Sex(M) x Sin (24 time) | -0.025 | | -0.261 | 0.215 |
|  |  | Sex (M) x Cos (24 time) | -0.717 | | -1.103 | -0.335 |
|  | Random | Nest (Intercept) | 32% | |  |  |
|  | (variance) | Sin (24 time) | 13% | |  |  |
|  |  | Cos (24 time) | 54% | |  |  |

The posterior estimates (medians) of the effect sizes with the 95% credible intervals (CrI) from a posterior distribution of 5,000 simulated values generated by the ‘sim’ function in R[1]. Variance components were estimated by the ‘glmer’ function for binomial errors with logit link function[2]. Variable ‘time’ was transformed to radians (2*time * π/period of interest: 24h) and fitted as sine and cosine of radians. Note that presented estimates are logit-transformed.

**Supplementary table S4** | **Circadian pattern of female exchange requesting**

|  |  |  | |  | 95% CrI | |
| --- | --- | --- | --- | --- | --- | --- |
| Response | Effect type | Effect | | Estimate | Lower | Upper |
| Exchange request effort | Fixed | Intercept | **-0.576** | | **-0.770** | **-0.387** |
|  |  | Sin (24 time) | **0.448** | | **0.297** | **0.593** |
|  |  | Cos (24 time) | **-0.607** | | **-0.902** | **-0.306** |
|  |  | Sin (12 time) | **0.230** | | **0.104** | **0.352** |
|  |  | Cos (12 time) | **-0.653** | | **-0.792** | **-0.515** |
|  | Random | Nest (Intercept) | 26% | |  |  |
|  | (variance) | Sin (24 time) | 9% | |  |  |
|  |  | Cos (24 time) | 65% | |  |  |

The posterior estimates (medians) of the effect sizes with the 95% credible intervals (CrI) from a posterior distribution of 5,000 simulated values generated by the ‘sim’ function in R[1]. Variance components were estimated by the ‘glmer’ function for binomial errors with logit link function[2]. Variable ‘time’ was transformed to radians (2*time * π/period of interest: 12 or 24h) and fitted as sine and cosine of radians. Note that presented estimates are logit-transformed. Estimates whose 95% credible intervals do not contain 0 are highlighted in bold.

**Supplementary table S5** | **Between nest differences in male contribution to incubation**

|  |  |  | 95% CrI | |
| --- | --- | --- | --- | --- |
| Response | Effect | Estimate | Lower | Upper |
| Proportion of male nest attendance | Intercept | **0.204** | **0.183** | **0.225** |
|  | Exchange request effort | 0.017 | -0.006 | 0.040 |
|  | Exchange request efficiency | **0.100** | **0.077** | **0.122** |

The posterior estimates (medians) of the effect sizes with the 95% credible intervals (CrI) from a posterior distribution of 5,000 simulated values generated by the ‘sim’ function in R[1]. Variance components were estimated by the ‘lm’ function in R. Both predictors were z-transformed (mean-centred and divided by SD). The model was weighted by square root of monitored time. Estimates whose 95% credible intervals do not contain 0 are highlighted in bold.

**REFFERENCES**

1. Gelman A, Su Y-S, Yajima M, Hill J, Pittau M, Kerman J ouni, et al. Data Analysis Using Regression and Multilevel/Hierarchical Models [Internet]. CRAN Repos. 2016. p. 1–53. Available from: https://cran.r-project.org/package=arm

2. Bates D, Maechler M, Bolker B, Walker S, Christensen RHB, Singmann H, et al. Fitting Linear Mixed-Effects Models Using lme4. J Stat Softw [Internet]. 2015;67:1–48. Available from: https://www.jstatsoft.org/article/view/v067i01
